# Ten simple rules for improving communication among scientists

**DOI:** 10.1371/journal.pcbi.1010130

**Published:** 2022-06-23

**Authors:** Carla Bautista, Narjes Alfuraiji, Anna Drangowska-Way, Karishma Gangwani, Alida de Flamingh, Philip E. Bourne

**Affiliations:** 1 Early Career Scientist Communication and Outreach Subcommittee, Genetics Society of America, Rockville, Maryland, United States of America; 2 Institut de Biologie Intégrative et des Systèmes (IBIS), Université Laval, Québec, Québec, Canada; 3 Département de Biologie, Faculté des Sciences et de Génie, Université Laval, Québec, Québec, Canada; 4 Regroupement Québécois de Recherche sur la Fonction, la Structure et L’ingénierie des Protéines (PROTEO), Université Laval, Québec, Québec, Canada; 5 Centre de Recherche en Données Massives (CRDM), Université Laval, Québec, Québec, Canada; 6 Department of Pharmacology, College of Medicine, University of Kerbala, Kerbala, Iraq; 7 Department of Biology, College of Arts and Sciences, University of Virginia, Charlottesville, Virginia, United States of America; 8 Department of Biology, University of Dayton, Dayton, Ohio, United States of America; 9 Carl R. Woese Institute for Genomic Biology, University of Illinois at Urbana-Champaign, Champaign, Illinois, United States of America; 10 School of Data Science, University of Virginia, Charlottesville, Virginia, United States of America; Carnegie Mellon University, UNITED STATES

## Abstract

Communication is a fundamental part of scientific development and methodology. With the advancement of the internet and social networks, communication has become rapid and sometimes overwhelming, especially in science. It is important to provide scientists with useful, effective, and dynamic tools to establish and build a fluid communication framework that allows for scientific advancement. Therefore, in this article, we present advice and recommendations that can help promote and improve science communication while respecting an adequate balance in the degree of commitment toward collaborative work. We have developed 10 rules shown in increasing order of commitment that are grouped into 3 key categories: (1) speak (based on active participation); (2) join (based on joining scientific groups); and (3) assess (based on the analysis and retrospective consideration of the weaknesses and strengths). We include examples and resources that provide actionable strategies for involvement and engagement with science communication, from basic steps to more advanced, introspective, and long-term commitments. Overall, we aim to help spread science from within and encourage and engage scientists to become involved in science communication effectively and dynamically.

## Introduction

Good communication enables the efficient and effective spread of ideas. Communication is a bilateral process by which 2 or more individuals enter into a transactional dialogue for the purpose of successful comprehension of content and intent to exchange messages sequentially for relative success [1]. Science communication is a broad term encompassing communication through multidisciplinary and interdisciplinary scientific teams in fundamental and applied science, education, community engagement, and outreach events that help raise science awareness. Science communication among scientists occurs through conferences and workshops, where scientific ideas are discussed, and questions on various research angles are answered. Communication in science also implies conventional networking activities such as collaboration between projects and multiauthored papers, activities which may help you establish a network of contacts. However, science communication also encompasses other more innovative areas such as scientific discussion forums, open-source software projects, or the contribution to scientific blogs. As a scientist in any field of Science, Technology, Engineering, and Mathematics (STEM), and at any level of education or career stage, these activities are essential for promoting fruitful discussions among peers, can encourage collaborations and research partnerships, and will help you get involved with your scientific community [2].

Participation in science communication activities can be rewarding. For example, it can enable new skills, expand one’s network, and be gratifying on a shorter timescale than research projects. However, advancement into a new era of communication where the pace at which information is shared and the number of collaboration opportunities have increased dramatically can be wholly overwhelming, especially to early-career researchers that are just starting their science communication journeys. Hence, science communication can also be time-consuming, lead to overcommitment, and cause delays to other competing priorities such as research projects. This article aims to provide early-career scientists and researchers with actionable ways and resources to engage in science communication effectively and dynamically while also respecting an adequate balance in the degree of commitment. Regarding the objective of this article, we focus on communication between scientists, specifically providing information about tools that spur effective communication. However, we believe that as scientists, it is very important to develop effective communication skills from an approach that also benefits the general public to ensure that science is accessible by everyone.

**Fig 1:**
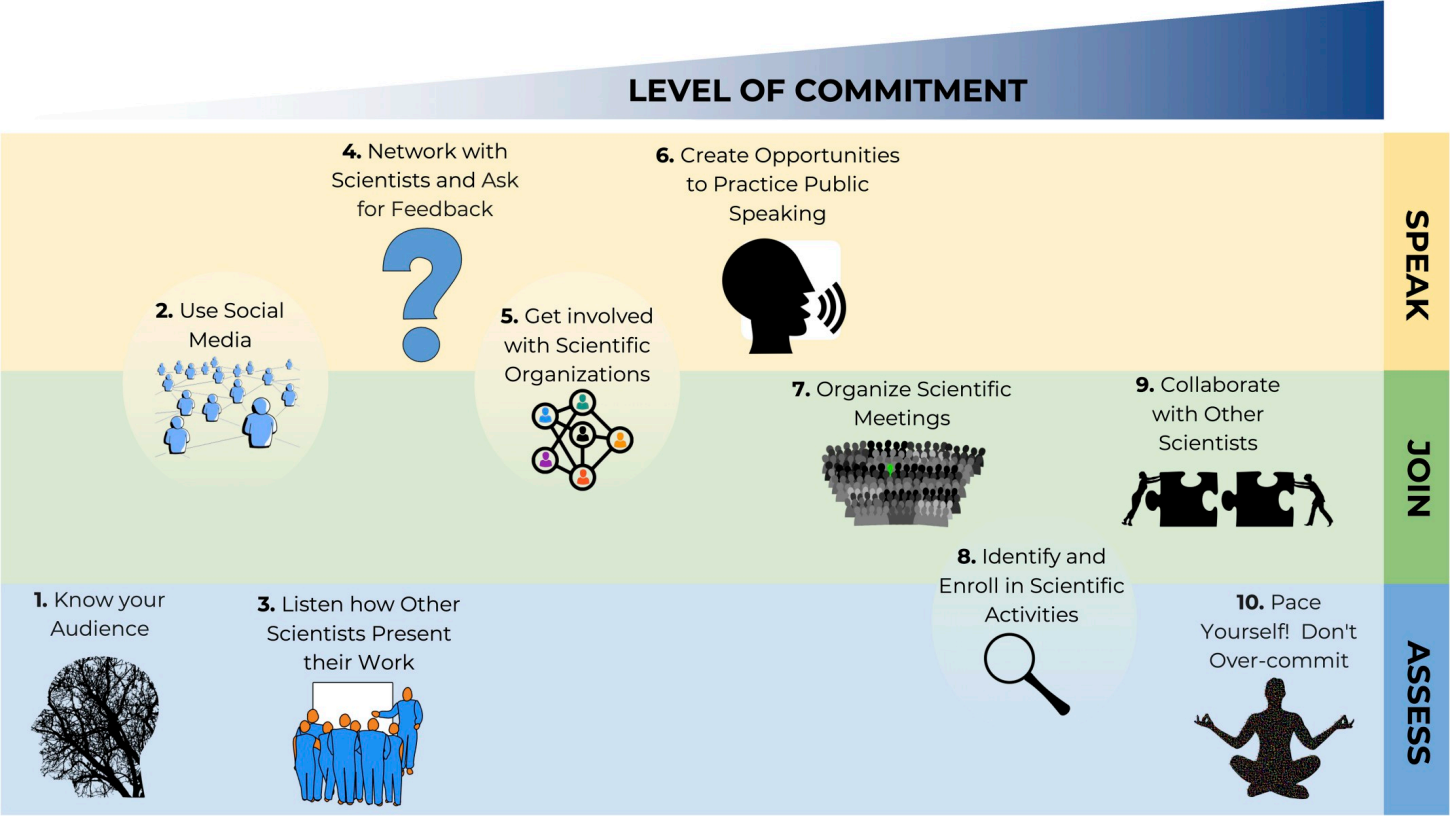
Outline of Ten simple rules for improving communication among scientists. These roles are organized based on the level of commitment required and grouped according to the categories: speak, join, and assess. The rules shown in circles form part of multiple categories. Figure created expressly for the article. Images contained within are from https://openclipart.org. Author credits are Rule 1: Gordon Dylan Johnson (@GDJ); Rule 2: Gordon Dylan Johnson (@GDJ); Rule 3: Bitterjug (@bitterjug); Rule 4: Priscilla (@barnheartowl); Rule 5: liftarn (@liftarn); Rule 6: amcolley (@amcolley); Rule 7: kattekrab (@kattekrab); Rule 8: Inkie 30 (@Inkie30); Rule 9: Gordon Dylan Johnson (GDJ); and Rule 10: Gordon Dylan Johnson (GDJ).

We developed 10 rules shown in increasing order of commitment, which are grouped into 3 categories (Fig 1): (1) speak, based on active participation that involves networking, getting feedback from other scientists as well as creating opportunities for public speaking; (2) join, based on joining scientific groups either in social networks, in scientific societies, in the organization of scientific meetings or collaborations with other scientists; and (3) assess, based on analyzing and listening to the way of communicating science of other scientists and identifying the necessary resources to improve your communication skills without overcommitting. Our approach is flexible as there are some rules that may form part of multiple categories. For example, Rule 2: Use social media (Fig 1) can form part of the speak category when actively engaging in Twitter dialogue, but could also refer to the join category when passively participating in or following Twitter posts or other social media. Although we’ve focused on science communication within science, the strategies and tools that we provide may also be helpful in other educational fields. Communication can take place in different forms and intensities, and we therefore frame our article to consider the time and level of effort required for various science communication activities and commitments. These rules stem from our own experiences as a part of the Early Career Leadership Program in the Communication and Outreach Subcommittee in the Genetics Society of America. We acknowledge that the listed levels of commitment (the order of Rules 1 to 10) might vary depending on effort and/or engagement (e.g., social media could include monthly tweets or, alternatively, could include multiple tweets per day and engagement on additional platforms). The levels of commitment may also vary in time commitment, where some tasks may require longer periods of time to complete (e.g., writing science communication articles or organizing outreach events), while others can be carried out quickly and/or while multitasking (e.g., listening to podcasts while preparing culture media in the laboratory or while commuting). We leave it to the reader to decide on their available time commitment and goals when choosing science communication engagements.

With these rules, we aim to provide scientists with actionable ways to exercise active, effective, and responsible communication among scientists that will help enhance their professional careers. Actionable tools and strategies such as those presented here may be especially helpful to early-career researchers. In addition, we refer to other “10 rules” articles as a resource list for exploration into specific topics that were only briefly mentioned as part of our rules. The list we provide here is nonexhaustive; however, we hope that these rules provide a useful tool kit for scientists to implement in the short and long term.

### Rule 1: Know your audience

Regardless of how science is communicated, the first question we should ask is who is the target audience? Who am I trying to reach? It is crucial to think about who your audience is and to tailor your science communication activities and engagements to fit the understanding and interests of the audience. What is interesting to one audience may not be of interest to others. Therefore, consider the audience’s interests and skills when you develop and present your research talk. By knowing your audience and their specific understanding of science or STEM, you can determine what terminology and tools are most effective for explaining concepts and communicating science. Within the scope of this article, science communication can be based on intraspecialist communication intended for specialists in the same field, but is also applicable to interspecialist communication between experts in different fields, communicating to decision-makers and businesses, or educational communication related to training and broader audiences (applied science communication accessible to broader audiences is often referred to as “Scicomm”).

In intraspecialist communication, experts in a particular specialization communicate using specific and complex terminology and they share research findings, debate, and discuss their science assuming that the target audience (other researchers in their field) will have adequate background knowledge to comprehend and engage with the material presented. If you are unsure of the extent of background the audience has, consider briefly introducing concepts, objectives, and important information that the audience should know before delving into your research. For interspecialist and other audiences, strive to explain your research in understandable and uncomplicated language. Using accessible language can lead to more citations of your research articles and encourage new collaboration opportunities. You might consider incorporating topics or concepts that can engage your target audience directly. To dive deeper into how to present your research project engagingly, we recommend reading Ten simple rules for the innovative dissemination of research [3]. Interspecialist science communication skills are emphasized in multidisciplinary research projects, internal communication at research institutions, educational purposes, and communication with decision-makers and business partners. Such skilled communicators often have thought and speech patterns typical of their discipline that have been honed through years of practice. Although science communication as part of research associated with multidisciplinary teams can be challenging, such skills can be improved with study and practice (see Rules 3, 4, and 9).

Many factors influence how effective and interpretable your science communication activities and engagements are, but paying attention to style and forum can help ensure that your message is delivered as effectively as possible. For example, writing a quick and concise message to a colleague in the laboratory through Slack (fast messaging commonly used in research teams) differs from talking with people from various research backgrounds at scientific conferences (more nuanced discussions are often required). Try to adjust your communication style to be formal or informal based on the type of audience with whom you are communicating. Similarly, identifying and understanding the target audience and forum can help you to focus on activities that promote the effective delivery of your message and may help encourage reciprocal engagement from the audience [4]. For example, you could ask rhetorical questions in a presentation or conduct live polls using online tools like interactive quizzes with Survey monkey, Kahoot, or Google Forms.

Regardless of whether we talk with experts or nonexperts, everyone loves a good story, and every scientist should strive to develop engaging storytelling skills so that they may communicate their own or other science well. However, discussions on how to frame your science communication and research as great/engaging stories is beyond the scope of this article. We recommend learning more about storytelling and developing an engaging narrative from Dr. Randy Olson’s books and workshops [5–7].

### Rule 2: Use social media

Social media can be a great way to interact with fellow researchers from all over the world. Social media platforms such as Twitter, Facebook, and LinkedIn have become a popular way to showcase, share, and promote science.

If you are unsure how to start your science Twitter account, have a look at these Ten simple rules for getting started on Twitter as a scientist [8], and start by following relevant hashtags, e.g., #AcademicTwitter or #PhDlife and others based on your specific science interests. By following relevant hashtags, you can find fellow scientists that are tweeting about topics that you are interested in, connect with other scientists in and outside of your field, and start building your network. Indeed, social media is becoming an important networking tool where conversations and interactions with other researchers can lead to new opportunities and collaborations. Some journals, for example, *Current Biology*, allow researchers to add their Twitter handles as a way for others to connect with authors. Similarly, many journals, including the preprint website bioRxiv, frequently publish information about new papers on their Twitter accounts, allowing easy access to new papers and research ideas by simply scrolling through your Twitter feed. Likewise, you can use Twitter as a tool to describe and promote your new articles, get feedback from peers, and you may achieve a greater reach than you would through traditional means of dissemination, e.g., at scientific conferences. Additionally, you may learn from and be inspired by science communicators such as @science.sam, @niniandthebrain, @sciencewithanni, or @sciencewhizliz to name a few. These are some examples that we personally found engaging and effective, but there are many others that you can find browsing through the hashtags!

Take advantage of the power of social media! For example, when you attend a scientific meeting, tweet about it! Do you not know where to start or how to tweet about conferences? You can use these Ten simple rules of live tweeting at scientific conferences [9]. You can also use social media to communicate your research to a broader audience and host online outreach events [10]. Last but not least, remember to adjust your language and approach to the specific platform, event, and audience that you are addressing.

### Rule 3: Listen how other scientists present their work

Listening to talks from other scientists can help you learn how best to communicate your research. Hosting webinars and talks on virtual platforms has become standard practice due to the Coronavirus Disease 2019 (COVID-19) pandemic and the associated travel and social distancing restrictions. Attending online talks may provide a cost-effective and accessible way (no travel required) to attend and listen to other scientists present their research, which can be beneficial for building confidence and experience so that you can effectively transition to in-person opportunities as they arise. Although presentation goals and topics may differ, some skills and techniques required to deliver good presentations are the same, whether online or in person. Watching talks outside of your scientific field may be especially helpful, as this will allow you to focus on how presenters explain difficult or complex topics in ways that are accessible to you.

The world’s most popular talk is by Sir Ken Robinson, “Do schools kill creativity?,” which has had almost 71 million views on the TED site (October 2021). Another successful speaker is the award-winning rocket scientist, self-help author, and host of the “Answers Unleashed” Podcast, Olympia LePoint. What do they have in common? They are able to keep a nonexpert public engaged when discussing difficult and complex topics. An easy way to benefit from observing others is to identify a talk that you find particularly interesting and take note of the speaker’s techniques and how they present complex scientific topics in an accessible way. You can ask yourself: Did the presenter give specific examples? Relate science to everyday life? Use humor to engage? How did the speaker organize the story they are telling? Write down and organize the techniques you respond to and think about how you can implement them yourself, and try them out the next time you give a talk. If you have a favorite speaker, think of the particular presentation style and actions that make that speaker stand out from the crowd. How do they engage and interact with the audience? What could you apply in your talks? Are there specific approaches or methods that the presenter uses, e.g., presentation structure, that could help transform complex and dense topics into understandable and exciting talks? [11]

Although learning from other scientists’ speaking styles is a key element here, presentation slides constitute a great means of communication and delivering information, whether designed for journal clubs, short conference talks, thesis committee meetings, or seminars. Multiple slides are usually built together to tell a story about a specific topic. Combining what is shown on the presentation slide with what is verbally presented in a complementary way is crucial. You can take advantage of other scientists’ presentations while attending scientific meetings, such as adopting ideas, managing presentation time, using headings, including essential points, referencing others’ work, ideas for good visualizations, avoiding cognitive overloading, and avoiding animations. The Ten simple rules for short and swift presentations [12] and Ten simple rules for making good oral presentations [11] are great resources to consider while preparing your presentation. Also, Ten simple rules for effective presentation slides [13] is an additional resource to consider as it details aspects of slide preparation and design elements for optimal slide effect and audience engagement.

Active engagement in scientific discussion is crucial for improving communication among scientists. Therefore, seek opportunities to engage the speaker (in public Q&A or during the coffee break) to discuss your shared interests directly while attending a conference or workshop. It is critical to develop and sustain an active and participatory listening habit.

Platforms such as TED talks, which feature subject area experts who have developed their communication skills to disseminate their science, are great platforms to listen to and learn from. Other platforms, such as podcasts, also provide efficient channels through which to communicate science. You can find a nonexhaustive list of recommended podcasts and other selected examples of public speaking resources in Table 1.

**Table 1:** Public speaking resources.

Source	Type	Description
TED	Virtual talks	Popular science talks
Nature Podcasts	Podcast	Research podcasts from *Nature*’s journalists and editors
LatinoLabs	Podcast	Day-to-day process of creating Science by Latino community
Bioscience talks	Interview series	Insights into the work of scientists behind outstanding stories
Inthosegenes	Podcast	Hip-hop–inspired podcast uncovering the lost identities of African descended Americans (Black culture)
Labroots	Webinar	Educational and research virtual events
Frontiers of Science	Webinar	Scientific discoveries, insights, and best practices

A nonexhaustive list of platforms that promote science communication and public speaking.

### Rule 4: Network with scientists and ask for feedback

Networking can help you establish collaborations that could lead to opportunities for the next step in your career, e.g., transitioning from graduate research to a postdoctoral position, and you may receive valuable feedback to improve your research and science communication skills. Interacting with scientists, whether at in-person or online conferences, seminars, scientific meetings, or through social media, is an important tool for expanding your scientific network. However, approaching renowned scientists who share our research interests can be intimidating and daunting. An excellent way to break the ice is to send an email indicating your interest in meeting with this person at a meeting that you will both attend. Think carefully about the objective of your email, for example, if you are looking for advice on your research or aiming to entice the person to attend one of your talks/poster presentations. Being brief and direct may help ensure that you receive a precise answer that fits your needs. Additionally, try to add a specific rationale for contacting them, so that the individual you are contacting fully understands the purpose of your message and feels directly involved in the conversation.

Conversations do not have to occur in a formal environment, e.g., you can approach someone during a coffee break and interact more informally. It is useful to prepare an “elevator pitch” of your research interests so that you can demonstrate your research potential in just a few words. Summarize your research’s most important points, including your objectives and, if possible, your salient results and their implications. Delivering your elevator pitch to the right contacts will allow you to express why you would like to connect and will also allow them to provide feedback on your project or presentation. We recommend structuring your elevator pitch in the And, But, Therefore (ABT) structure. To learn more about ABT, we refer you to Dr. Randy Olson’s books [5,6].

In addition, when connecting with scientists that you are specifically interested in talking to, with the prospect of establishing a long-term acquaintance, make sure to review their publications and familiarize yourself with their overall research portfolio, place of work, and anything else that might spark a memorable conversation.

Finally, capitalize on the power of social media software applications, such as Twitter, Facebook, or LinkedIn. Social media enables quick and effective connections with scientists from all over the world (see Rule 2). Regardless of how you initially connect, keeping in touch after the fact could help reinforce the connection, promote future interactions, and encourage collaborations within your scientific field. Sending a follow-up email or a direct message on Twitter thanking a person for their time can help them remember you in the future and provides a way for them to get in touch with you.

### Rule 5: Get involved with scientific organizations

Formal and informal scientific organizations play a vital role in enabling and promoting science by bringing together scientists from complementary scientific fields, advancing science globally, enhancing communication among scientists worldwide, and public engagement with science. Some organizations offer programs that specifically aim at developing and promoting leadership and communication for early-career researchers to sharpen their skills in career development, communication, community engagement, and outreach. These include, but are not limited to, the Genetics Society of America’s Early Career Scientist Leadership Program, The American Society for Cell Biology’s Committee for Postdocs and Students (COMPASS), eLife Ambassador’s Program, or even parallel programs that aren’t organized around disciplinary areas, such as SACNAS. Similarly, you can also participate locally in scientific groups or student committees in your city.

Being an active member of a scientific society can help you build your network and hone your skills to boost and support your future career. If you aim to sharpen your science communication skills in particular, you can join a committee that focuses specifically on science communication and outreach. Such committees often write science pieces for broad audiences [14] or for fellow scientists, e.g., through outreach activities on broader public platforms such as Massive Science, ecrLife, Science Trends, The Conversation, Discover Magazine, WIRED Science, etc. Partaking in groups that focus on science communication can help you become proficient in writing for diverse target audiences, from newspapers or printed media to scientific publications and grant applications. You can also develop workshops, panels, and meetings where topics that are important to a given community can be discussed. Moreover, joining scientific communities allows you to contribute and help address the community’s needs. By being an active member of a scientific society, your voice can be heard, and your opinion is considered while making decisions regarding the values and priorities of the scientific organization.

### Rule 6: Create opportunities to practice public speaking

Scientists communicate about their research throughout their careers. Learning how to give talks of different lengths and for a variety of audiences is an essential skill. Many platforms offer different types of talks for diverse audiences (e.g., the general public or more specialized audiences) and environments (e.g., academic or less formal interactions). Practicing your public speaking with diverse audiences and settings will teach you to adapt your presentation style and goals for each public speaking engagement. The presentation format is also essential; for example, poster presentations are generally more interactive and a presenter might be stopped and asked questions providing more room for discussion [15].

Practicing to communicate with broader audiences [16] and communicating your topic without jargon will improve communication with fellow scientists (see Rule 4), especially with those outside your field of study. Therefore, aim to find places (or organize them yourself, see Rule 7) where you can practice presenting longer 1-hour talks [17] or shorter 15-minute seminar-style presentations [12]. Similarly, you can prepare a “poster pitch” to engage people during your poster session at a conference. Having short and long versions of your presentation handy may be very useful depending upon a person’s interest level. Furthermore, lab meetings and journal clubs are great settings to work on communication skills you’ve learned in other talks (see Rule 3) in a relaxed environment. You can also try Skype a Scientist, which connects scientists worldwide with teachers, classrooms, or groups. Another option is to practice talks in more relaxed environments, such as a bar or other social environment. Practicing in informal settings can help you gain the skills to communicate more easily and comfortably in formal settings. Pint of Science, for example, organizes science talks at breweries. You can also participate in Science Clubs where scientific engagement with the youth is promoted. Finally, do not hesitate to ask your family and friends for feedback. They can give you very useful advice related to public speaking. A great starting point for practicing talks is your lab meeting and journal clubs often organized by the departments. When it is your turn to present your research to your lab or department, prepare well and use this opportunity to improve your skills. Finally, as mentioned in Rule 10, it is important that you choose these opportunities carefully and that you only prioritize opportunities that can help you the most in each phase of your career (Table 2).

**Table 2:** Activities according to the stage of the scientific career.

	Graduate student (early years)	Graduate student (advanced years)	Postdoc	Faculty
**Social media**	- Create an account on one social media platform - Start posting regularly (e.g., once a week) about your topic of interest	- Interact with PIs whose labs you are interested in joining as a postdoc - Be on the lookout for their postdoc offers	- Keep being consistent with your posts and interact with your peers - Highlight your papers and disclose its scope	- Create a social media account for your laboratory team - Post the achievements of lab members and activities you do as a lab - Post graduate and postdoc positions to reach international candidates
**Networking and collaboration**	- Attend seminars within your institution - Practice networking in your department with your peers and professors	- Attend local, national, and international conferences in your field - Capitalize on your network to find a postdoc in academia (for positions outside of academia network through, e.g., LinkedIn)	- Present your research at international conferences - Always ask for feedback on your research, and participate in Code Review - Look for collaborations	- Keep attending conferences and give talks at various institutions - Talk with many scientists, also outside of your immediate field to build new collaborations
**Outreach**	- Participate in workshops related to science communication	- Write a few outreach articles for a broad audience (3 to 4 a year)	- Join initiative that aims to change a policy that is within your field of expertise	- Consider writing a book about your research topic directed to broad audiences
**Scientific organizations**	- Join a local/university group within your topic of interest	- Join an international group of scientists that is related to your field	- Take on a leadership role in a group that is part of a science organization	- Become an advisor or mentor to a group associated with international scientific organizations
**Leadership**	- Organize a student-oriented scientific conference in your institution	- Organize local conferences - Organize a workshop in a national conference	- Help organize a national conference on your research topic	- Organize an international conference in your field (e.g., FASEB Catalyst Conference or Science Research Conference)
**Public speaking**	- Make the most of opportunities to give talks at your department seminars and lab meetings	- 3 Minutes Thesis competition - Give a talk or present a poster at a bigger conference - Once a year give a talk at a local student conference	- Give a talk to a broad audience at least once every 6 months - Pint of Science - Once a year give a talk at a local conference	- Make sure your lab has regular lab meetings - Give students constructive tips on how to improve their presentation skills - Give a talk at a bigger conference once a year

A nonexhaustive list of activities combining the above rules that a scientist can carry out during the different stages of the scientific career.

### Rule 7: Organize scientific meetings

Have you ever considered organizing a scientific meeting or a conference? The organization of these events will help you hone leadership skills and expand your network, which can help you develop skills that may be valuable throughout your career. As you interact firsthand with the presenters, you can gain organizational and project management skills and learn what is needed to set up successful events.

Great, but how should I start? The first trick is to ensure that you are not overwhelmed; start small, organize local meetings, for example, student seminars, colloquia, or symposia in the institutional department of your university, and call on others to help. Providing opportunities to your peers (e.g., other early-career scientists) to join you in organizing meetings can help you share this valuable skill set, thereby benefiting them too, but will also allow you to share responsibilities and commitments so that you are not overwhelmed. You can begin by inviting only students and later expand your audience by inviting established scientists, first within and later outside your institution. The theme or topic of the event is critical as it will determine your audience. Look for a topic that is not only related to your research but also your research center, institute, or broader academic field of study. To get started, you might express your intention and willingness to organize events at your institution—maybe there is already an existing trainee committee that you can join?—and start inviting guest speakers that you find interesting via email (see Rule 4); this can help you network with scientists from within your comfort zone: your institution. You can learn how to interact with other peers and get the most out of these interactions by participating in the scientific discussion.

Additionally, you will likely come across activities of interest to you that you can organize in your institution, center, or even with your research group:

- **Journal clubs:** You can periodically meet with scientists and plan to discuss a technique, biological topic, or classic papers’ series. It is an easy way to keep up to date with the cutting-edge scientific literature relevant to your research topics and those of your colleagues and peers. Additionally, you can invite authors to discuss the article with you to improve successful networking.
- **Bioinformatics clubs:** You can dedicate time to follow the advances of bioinformatics [18], for example, data visualization.
- **Book clubs:** This is similar to a journal club, but you may focus on discussing an entire book. This type of club allows more general learning, and since it might cover more general topics, it can help you connect with people in different (but related) fields.

If you want to think bigger, you could expand your meeting to connect with regional or international research groups or institutions. Conferences often allow early-career scientists to organize workshops (The Allied Genetics Conference, Gordon Research Conferences, etc.). You can assemble a team of interested colleagues and submit a proposal for a workshop at an international conference. It is a unique opportunity to reach a wider audience, and such opportunities allow you to showcase your research and make yourself known outside the borders of your institution or country and may allow you to connect with international scientists in your field. Ten simple rules for organizing a data science workshop [19] provide valuable tips that might be relevant when you start planning a workshop for the first time.

Finally, keep in mind the utility and accessibility of virtual events. The COVID-19 pandemic has led to many events transitioning from in-person to online platforms; therefore, developing online science communication skills is critical to master. Familiarize yourself with commonly used virtual platforms (Zoom, Teams, etc.) and other more interactive platforms widely used in conferences (Gather, Online Town, Discord, etc). In addition, we strongly recommend following the Ten simple rules for organizing a non–real-time web conference [20]) and Ten simple rules for organizing a webinar series [21] that will be useful for transferring your activities to an online format.

### Rule 8: Identify and enroll in scientific activities

Although analyzing what resources you need and the resources that could benefit your career is arduous and time-consuming in light of the rules discussed, there is no need to reinvent the wheel. If you are interested in science communication opportunities, e.g., assisting scientific events, an achievable start is to search for available opportunities within your institution or scientific field and start from there. Look for opportunities to participate in organizing established events. Many institutions support science communication by providing specialized courses (e.g., University of Chicago) or science communication support (e.g., Newcastle University) to help scientists communicate research to diverse audiences using appropriate platforms.

Nevertheless, if your institution does not offer these opportunities, you do not have to start from scratch. Reach out to your institution, school, or department and propose the organization of activities you are interested in journal clubs, book clubs, etc. (see Rule 7). You can also start via a faculty sponsor, such as a lab PI or instructor of a course you enjoyed by communicating your interest in carrying out these activities. Prior experience as a science communicator is not required; seeking advice and adapting a template from similar activities available at your institute is helpful when you are an early-career scientist with limited experience. Additionally, you can join and actively participate in online forums that may provide resources without the requirement of a time commitment and from which you can obtain useful skills for your future: science clubs, online outreach activities (see Rule 3), or you can even start your own podcast.

### Rule 9: Collaborate with other scientists

Sometimes, we find ourselves at a crossroads in our research. Identifying problems and finding solutions is part of every scientist’s career. Nevertheless, you should also be able to ask for help. Suppose a part of your project extends beyond your knowledge base or beyond that of your colleagues within your school or institution. In that case, you might identify another team of researchers that has the expertise you need. You can write to them directly and benefit from their advice and/or start a collaborative project. Collaboration with other researchers is essential, especially for interdisciplinary research. Asking for help or feedback from experts on a topic that you are less familiar with can be productive and beneficial. In the context of this article, we view science collaboration as a flexible and encompassing term: interacting and working with other scientists and stakeholders to discuss, conduct, and disseminate research both formally in academic settings, but also on informal platforms (e.g., social media).

Collaboration can happen in very diverse ways: from innovative activities by participating in open forums for scientific discussion (e.g., Reddit Science) or in open-source software projects (e.g., Julia project), to more conventional scientific activities such as writing grant proposals, reviews, and articles with specialists in the fields of your interest, lending your expertise on research projects, or even sharing hypotheses and methods that have worked well in your experiments. Starting a scientific collaboration might involve more significant effort but may be worth it as it increases your visibility, leadership, and organizational skills.

However, it is essential to set clear expectations when initiating collaborations: Carefully choose the collaborations that fit your objectives and ethics and prioritize effective communication with and within your team [22]. Also, consider and choose collaborations that fit within your available level of commitment (see Rule 10). Level of commitment may vary with the depth and breadth of collaborative interaction; for example, whether you are leading a project or contributing as a participant may require different efforts and, similarly, whether there are a few or many collaborators that are contributing to a project may also impact your own level of commitment. Consider, for example, which collaborations best fit your current availability and scope:

- collaboration within your institute (to share some techniques, ideas, concepts, etc.) and
- collaboration bridging institutes (from networking at conferences, stays in foreign laboratories, etc.).

Collaborations or research partnerships may lead to writing a multiauthored paper. For successful collaborative writing, you must establish a clear writing strategy that considers and allows for the contribution of all members, choose online tools that fit your needs both when writing and when sharing data, and, finally, always be transparent and agree on the authorship based on contribution [23,24].

### Rule 10: Pace yourself! Don’t overcommit

Time is a limited resource. This article presents you with different strategies to improve your communication with other scientists, but we do not expect you to commit to all of them. Before committing, make sure that you have enough time in your schedule. Be honest with yourself in this process. It is sometimes difficult to refuse an opportunity, but it is better to pass on it than to overcommit. Overcommitting can lead to burnout while trying to complete all of the tasks on your to-do list. In academia, overcommitment has been shown to have implications for mental health, job satisfaction, and job retention [25].

To protect yourself against the negative personal [26] and professional [27] impacts of overcommitment,

- aim to establish effort–reward balance for projects and collaborations that you commit;
- try to assess which activities are most important to you and that will advance your career in the direction you want to go (e.g., academia or industry); and
- commit to those and politely decline the others.

Another way to avoid overcommit is to carefully choose the activities that most benefit each stage of your career. Many of the activities that we list can be combined, so you can gain the benefits of multiple rules by being involved in one activity. We give you a few examples of activities you can perform in different stages of your scientific career (Table 2). The list is not exhaustive, but provides an idea of the activities available for scientists combining the rules that were mentioned throughout the article.

Being able to set your priorities is one of the most valuable skills you can learn. You may be amazed that you can achieve more by taking on less and that quality of your work will trump the quantity. If you have trouble deciding, we recommend you try doing a Productivity Purge as described in Professor Cal Newport’s blog.

## Conclusions

Communication among scientists is crucial because it allows for scientific cohesion and the advancement in knowledge. However, learning how to communicate science effectively is not an easy task. Therefore, to succeed, scientists must learn how to communicate effectively and dynamically, regardless of their field or career stage and/or career path. We, as scientists, need to convey our messages clearly and coherently so that other scientists and other audiences are engaged, aware of and remember our research findings. This article highlighted the importance of communication within science and ways to improve communication skills, with a particular focus on rules that provide tools that may benefit early-career scientists. We provide a general overview of these strategies through these 10 simple rules and encourage scientists of all career stages to get involved in science communication. As a researcher, you can interact with your fellow scientists through social media to build collaboration and announce scientific activities. Conferences, seminars, and research meetings provide excellent opportunities for giving and receiving feedback. As a speaker, identify your strengths and weaknesses and improve your skills over time by looking for opportunities to practice public speaking. Participating in collaborative writing (e.g., grant proposals, review articles, or research papers) unites scientists to share their expertise and expand their network. Joining scientific societies and participating in science-orientated outreach are good opportunities to improve your communication writing skills while raising awareness about science.

Here, we provide 10 simple rules to help you start and/or sustain your journey as a science communicator. However, we realize that this list is nonexhaustive and that there are many opportunities and modes of science communication that are beyond the scope of this article. For example, honing formal and informal writing skills, including language and grammar, can be critical for effectively and efficiently communicating your science and research. You can find help with academic or formal writing by following, e.g., these Ten simple rules for writing research papers [28] and these Ten simple rules for scientists: Improving your writing productivity [29]. For help with less formal writing, consider following e.g., these Ten simple rules for writing a popular science book [30], or branch out and consider alternative modes of communication, e.g., by following these Ten simple rules for drawing scientific comics [31].

Involvement in activities that improve science communication requires time, dedication, and consistent efforts. However, these efforts have professional payoffs, not only in improving communication skills but also by helping you gain leadership, mentoring, and organizational skills. Thus, choose the activities you can commit to efficiently and according to your availability; overcommitment could result in poor performance or anxiety. Commitment to science communication is a voluntary activity, and it requires dedication. However, keep in mind that even small contributions can make a significant impact. Here, we have presented rules that can be advantageous on a professional level, but science communication also benefits society at large. Being a diligent and dedicated researcher can make you a great science advocate, can allow you to interface with and communicate accessible science to the public, can help you contribute to other people’s scientific journeys, and can help you to feel more fulfilled by being part of a community.
